# Associations of physical activity with depressiveness and coping in subjects with high-grade obesity aiming at bariatric surgery: a cross-sectional study

**DOI:** 10.1186/s13030-015-0042-4

**Published:** 2015-06-25

**Authors:** Ulf Elbelt, Anne Ahnis, Andrea Riedl, Silke Burkert, Tatjana Schuetz, Juergen Ordemann, Christian J. Strasburger, Burghard F. Klapp

**Affiliations:** Department of Endocrinology, Diabetes and Nutrition, Charité - Universitätsmedizin Berlin, Charitéplatz 1, 10117 Berlin, Germany; Charité Center for Internal Medicine and Dermatology, Division of General Internal and Psychosomatic Medicine, Charité - Universitätsmedizin Berlin, Charitéplatz 1, 10117 Berlin, Germany; Institute of Medical Psychology, Charité - Universitätsmedizin Berlin, Luisenstr. 57, 10117 Berlin, Germany; IFB Adiposity Diseases, Leipzig University Medical Center, Philipp-Rosenthal-Str. 27, 04103 Leipzig, Germany; Department of Surgery, Obesity Center, Charité – Universitätsmedizin Berlin, Charitéplatz 1, 10117 Berlin, Germany

**Keywords:** Bariatric surgery, Coping, Depression, Obesity, Physical activity

## Abstract

**Background:**

Reduced physical activity is supposed to be associated with depressiveness and more passive coping patterns. For further evaluation of this assumed relation we studied energy expenditure due to physical activity - usually referred to as activity thermogenesis (AT) - together with depressiveness (clinical diagnosis, depression module of the Patient Health Questionnaire), and coping behaviours (Brief COPE Inventory) in 50 patients with high-grade obesity (42 ± 12 years; 9 with II° and 41 with III° obesity) aiming at bariatric surgery.

**Methods:**

AT was assessed with a portable armband device (SenseWear™ armband). Depressiveness and coping were assessed using validated questionnaires.

**Results:**

Weight-adjusted non-exercise AT and intensity of physical activity (metabolic equivalent) correlated inversely with body mass index (non-exercise AT: *r* = −0.32, *P* < 0.05; mean metabolic equivalent: *r* = −0.37, *P* < 0.01) but not with depressiveness. The coping strategies “support coping” and “active coping” showed significant inverse correlations to a) weight-adjusted non-exercise AT (“support coping”: *r* = −0.34, *P* < 0.05; “active coping”: *r* = −0.36, *P* < 0.05), b) weight-adjusted exercise-related AT (“support coping”: *r* = −0.36, *P* < 0.05; “active coping”: *r* = −0.38, *P* < 0.01) and c) intensity of physical activity (for mean metabolic equivalent: “support coping”: *r* = −0.38, *P* < 0.01; “active coping”: *r* = −0.40, *P* < 0.01; for duration of exercise-related AT: “support coping”: *r* = −0.36, *P* < 0.05; “active coping”: *r* = −0.38, *P* < 0.01).

**Conclusions:**

AT was not associated with depressiveness. Furthermore, supposed adaptive coping strategies of individuals aiming at bariatric surgery were negatively associated with AT.

## Background

Obesity is mainly caused by an imbalance of energy intake and energy expenditure [1] and leads to increased morbidity and mortality [2, 3]. Total energy expenditure derives from resting energy expenditure which accounts for approximately 60 % of it in sedentary or obese individuals [4], energy expenditure due to physical activity and the thermogenic effect of food. Energy expenditure due to physical activity is usually referred to as activity thermogenesis (AT). Higher levels of physical activity leading to increased AT have been shown to improve weight loss and to be a good predictor of long-term weight loss maintenance in obese individuals [5]. AT can be further divided into exercise-related AT and non-exercise AT [4]. Of special interest is non-exercise AT (NEAT), i.e. AT during low- and moderate-intensity physical activities, as it is reported to be the most varying component of energy expenditure [6] and it is the quantitatively more significant component of AT [7, 8]. In a prospective study lower levels of non-exercise AT were shown to play an important role in the pathogenesis of obesity [8].

Higher body mass indices [9–12] and increased visceral fat [13–15] are frequently reported to be associated with depression or depressive symptoms, e.g. in a large state-based telephone survey in the US [9], adults with current depression (assessed in a telephone interview with the Patient Health Questionnaire 8 [PHQ-8]) or a history of diagnosed depression or anxiety by health care professionals were shown significantly more likely to be obese and physically inactive than those without this diagnosis [9]. Additionally, improvements in mood disturbance scores (assessed with the Profile of Mood States Short Form: negative mood, depression and tension) were associated with higher weight loss by severely obese women participating in a cognitive-behavioural exercise support treatment [16]. In another study achievement of a weight loss of 5 kg or more is reported to be more likely for obese women with symptoms of depression, if they are enrolled in a behavioural treatment program focusing on both weight loss and depression; this study showed an association of improvement in depression with an increase in self-reported physical activity [17]. In line with these findings, negative correlations of depression or depressive symptoms with physical activity [9, 11, 18, 19] or physical activity self-efficacy (i.e., an individual’s confidence in his or her ability to manage physical activity behaviours) [10] have been reported. In addition, increases in physical activity proved protective against the onset of depressive symptoms [20, 21] and beneficial effects of physical activity on the course of depression have been described [17, 22, 23]. Therefore, physical activity is regarded as a preventive as well as a therapeutic treatment option in both conditions - obesity and depression [24], resp. their co-morbidities.

The multimodal approaches for the *conservative treatment* of obesity (change in diet, modification of eating behaviour, increase of physical activity, cognitive behavioural therapy, relaxation techniques) focus on behavioural changes of obese individuals for achieving long-term weight loss [25]. Therefore, strategies of coping - commonly defined as cognitive and behavioural efforts used to manage external and internal demands [26] - play a central role in the treatment of obesity. In particular, obese individuals do not only have to cope with physical and mental consequences of obesity itself and with challenges of weight reduction attempts, but also with negative or discriminative social issues like stigmatization (obesity bias) [27]. However, for individuals with a body mass index (BMI) above 40.0 kg/m^2^ or with a BMI between 35.0 and 39.9 kg/m^2^ and co-morbidities such as type 2 diabetes and other metabolic disorders *bariatric surgery* is the most effective treatment in terms of long-lasting weight loss [25].

Coping includes strategies that help the individual to feel better, regardless of whether these strategies are positively adaptive (problem focused) or potentially dysfunctional [28] with respect to objective changes like weight loss. Successful behavioural factors for the achievement of long-term weight loss, like more initial weight loss, reaching the self-determined weight goal, gaining a physically active lifestyle, achieving a regular meal rhythm inclusive breakfast and healthier eating, control of over-eating, and self-monitoring of behaviours are listed in a comprehensive review by Elfhag and Rossner [29]. For successful maintenance of achieved weight loss psychological coping strategies must encompass high levels of self-efficacy, effective problem-focused coping strategies with stressful situations and the ability to generate coping responses for anticipated high risk scenarios (for relapse of overeating or physical inactivity) [30]. In contrast, weight re-gainers frequently report “escape-avoidance” coping such as eating, sleeping or wishing the problem would go away [30]. Rydén *et al.* [31] studied obesity-related coping in relation to treatment preference (surgery versus conventional treatment) in patients enrolled in the Swedish Obese Subjects intervention study. In their cohort of 2510 patients surgical candidates displayed lower scores for problem-focused (adaptive) coping (“social trust”, “fighting spirit”) and higher levels of emotion-focused (maladaptive) coping (“wishful thinking”) than patients intending conventional therapy for weight loss [31].

Detailed information on direct relations of AT with depression, depressive symptoms, or coping styles of individuals with high-grade obesity aiming at bariatric surgery is not available, most likely due to difficulties in assessing AT and physical activity patterns precisely [32, 33]. However, modern technical devices like the SenseWear^TM^ PRO 3 armband (BodyMedia, Inc., Pittsburgh, PA, USA) allow more reliable assessment of energy expenditure and physical activity patterns under ambulatory conditions [7, 34, 35].

In consequence of our previous finding that AT and intensity of physical activity gradually decline with rising weight categories from normal weight to subjects with III° obesity [7] we also assumed that within a cohort of obese individuals intending bariatric surgery AT would be more reduced and physical activity patterns would be further altered with increasing of obesity. We hypothesized that this assumed reduction of AT and physical activity would be correlated with the degree of depressiveness and a dominance of passive (maladaptive) coping patterns.

## Methods

### Participants

Fifty outpatients (10 men, 40 women, see Table 1) who presented for evaluation prior to bariatric surgery between spring 2008 and summer 2010 at Charité - Universitätsmedizin Berlin by the Divisions of Clinical Endocrinology and General Internal and Psychosomatic Medicine were included in the study. The participants had a mean age of 42 ± 12 years (range 22–72 years). A medical history was taken and all participants underwent a complete physical examination. Weight was measured to the nearest 0.1 kg and height to the nearest 1 cm. BMI was calculated as kg/m^2^. The severity of obesity was classified according to the WHO criteria. Nine participants suffered from obesity II° (BMI 35.0–39.9 kg/m^2^) and 41 participants from obesity III° (BMI ≥ 40.0 kg/m^2^). Endocrinological evaluation showed three patients suffering from subclinical (mild) hypothyroidism. However, another 15 participants were on replacement therapy with levothyroxine due to autoimmune hypothyroidism. One participant was treated with oral glucocorticoids due to a pseudolymphoma of the orbit leading to exophthalmos; clinical history and cortisol excretion did not indicate the presence of Cushing’s syndrome in any of the patients. A psychosomatic evaluation (which in Germany is mandatory for cost coverage of bariatric surgery by the patients’ health insurance companies) with the purpose of identifying factors opposing bariatric surgery or determining an appropriate point in time for bariatric surgery was performed and a set of questionnaires measuring psychological variables, like depressiveness and coping mechanisms, was administered on a personal digital assistant. Participants suffering from diseases hindering normal daily physical activities such as clinically relevant heart failure, limiting pulmonary diseases, severe osteoarthritis or amputations were not included in the study. All patients provided written informed consent for the scientific use of their data.

**Table 1:** Demographic data, activity thermogenesis and patterns of physical activity

	Obesity		*P*	Depression		*P*	Replacement therapy with levothyroxine		*P*
	II° (*n* = 9)	III° (*n* = 41)		No (*n* = 32)	Yes (*n* = 18)		No (*n* = 35)	Yes (*n* = 15)	
*Demographic data:*									
Age, mean years (±SD)	46 ± 16	42 ± 11	Ns	41 ± 13	46 ± 9	Ns	42 ± 11	43 ± 14	Ns
BMI, mean kg/m^2^ (±SD)	38 ± 1	48 ± 6	*	46 ± 6	46 ± 7	Ns	46 ± 6	46 ± 8	Ns
*Activity thermogenesis:*									
NEAT/kg, mean kcal/d (± SD)	8.3 ± 3.1	4.9 ± 2.4	*	5.3 ± 2.2	5.7 ± 3.7	Ns	5.6 ± 3.0	5.3 ± 2.3	Ns
EAT/kg, median kcal/d (quartiles)	1.3 (0.7/2.4)	0.4 (0.1/1.3)	Ns	0.8 (0.3/1.4)	0.3 (0.0/2.1)	Ns	0.5 (0.1/1.4)	0.4 (0.0/2.2)	Ns
*Physical activity:*									
Steps/d, mean (± SD)	7750 ± 2153	5867 ± 2688	Ns	6023 ± 2459	6532 ± 3085	Ns	6275 ± 2712	6044 ± 2695	Ns
MET, mean (± SD)	1.23 ± 0.16	1.03 ± 0.16	*	1.09 ± 0.16	1.04 ± 0.20	Ns	1.08 ± 0.18	1.05 ± 0.17	Ns
Duration of EAT, median min/d (quartiles)	15 (7/21)	5 (1/13)	*	8 (3/15)	3 (0/17)	Ns	5 (2/15)	5 (0/20)	Ns

*BMI* body mass index, *EAT/kg* exercise-related activity thermogenesis/kg body weight, *MET* metabolic equivalent, *n* number of participants, *NEAT/kg* non-exercise activity thermogenesis/kg body weight, *Ns* not significant, *SD* standard deviation, *: *P* < 0.05

Mean and standard deviation are given for parametric data, median and quartiles are given for non-parametric data. Two-tailed student t-tests for parametric data and Mann–Whitney-U-tests for non-parametric data were used

### Registration of energy expenditure and patterns of physical activity

The SenseWear^TM^ PRO 3 armband (BodyMedia, Inc., Pittsburgh, PA, USA) utilizes a multi-sensor array including a 2-axis accelerometer, heat flux sensor, galvanic skin response sensor, skin temperature sensor and a near-body ambient temperature sensor for the estimation of total energy expenditure (TEE) and physical activity. Furthermore, with this device the degree of physical activity can be assessed and classified as either exercise-related AT (EAT) or non-exercise AT (NEAT) [7]. Therefore, we take the portable armband device for a suitable tool for the measurement of the different components of TEE. Data were analysed using a generalised proprietary algorithm developed by the manufacturer (InnerView™ Professional, Version 6.1, BodyMedia, Inc., Pittsburgh, PA, USA).

Participants were asked to wear the armband at least 20.5 h per day. Physical activity was registered for three days (preferably 2 weekdays and 1 weekend day) with the portable armband device. While total energy expenditure (TEE) was directly received from the proprietary algorithms of the portable armband device, activity thermogenesis (AT) had to be calculated according to the equation: AT = total energy expenditure (TEE) – thermic effect of food – resting energy expenditure. The thermic effect of food was estimated with 10 % of TEE and was calculated as TEE × 0.10 [4]. Resting energy expenditure was calculated according to the equation of Müller *et al.* for obese individuals [36]: Resting energy expenditure (MJ/d) = 0.05 x weight (kg) + 1.103 × gender – 0.01586 × age (years) + 2.924 (for gender: female = 0 and male = 1). Then resting energy expenditure was converted to kcal/d.

In addition, the number of steps, metabolic equivalents (MET), and the duration of activity thermogenesis with distinct levels of physical activity graded in MET were directly obtained from the proprietary algorithms. MET define the energy expenditure related to body weight. One MET is equivalent to 1 kcal/kg body weight/hour. *Energy expenditure of > 5 MET was classified as exercise-related AT (EAT). Energy expenditure of ≤ 5 MET was classified as non-exercise AT (NEAT)* [7]. Lastly, NEAT was calculated as AT – EAT.

### Assessment of psychosocial dimensions

As part of our psychosomatic assessment participants were individually interviewed *with special attention for the presence and the degree of depressive symptoms* by an experienced clinician. Depression was clinically diagnosed according to ICD-10 (International Classification of Diseases 10th Revision) by the interviewer and in case of diagnostic uncertainty after discussion by the psychosomatic team. In addition a set of questionnaires was presented with personal digital assistants (PDA):

*Depressiveness* In addition to the clinician’s diagnosis, depressiveness was assessed with the 9-item depression module from the Patient Health Questionnaire (PHQ) which is a well validated and widely used screening instrument [37]. Sum scores may range from 0 to 27 with higher values indicating higher levels of depressiveness. The ICD-10-Symptom-Rating questionnaire (ISR) consists of 29 items based on relevant symptoms for the assessment of psychological disorders according to ICD-10 [38], as well as a “total score”. The six syndrome scales include “depressive syndrome”, “anxiety syndrome”, “eating disorder syndrome”, “obsessive syndrome”, “somatoform syndrome”, and a “supplementary scale”, which covers a miscellaneous variety of syndromes. The ISR aims at a comprehensive evaluation of the severity of each disorder. Scores for the subscales and the total score range from 0 to 4 with higher values indicating a more severe syndrome [39].

*Coping strategies* Coping strategies were assessed with the German version of the Brief-COPE which consists of 28 items in four scales: “avoidant coping”, “support coping”, “positive reframing” and “active coping” [28]. This self-reporting questionnaire measures coping and explores strategies of handling difficult or awkward situations. Sum scores range from 6 to 24 for the scales “avoidant coping”, “support coping”, and “positive reframing”. For the scale “active coping” it ranges from 4 to 16.

*Assessment of further psychosocial dimensions* Although our primary focus was the assessment of depressiveness and coping strategies we aimed at a more comprehensive psychosocial characterisation of our patients. Therefore, in addition, anxiety, physical complaints, and quality of life and mood were assessed. These instruments also served for testing the plausibility of clinical diagnosis of depression.

*Anxiety* Anxiety is frequently associated with depression and can often be considered as a symptom of depression [40]. The 7-item self-report Generalized anxiety disorder scale (GAD-7) was used [41], a well validated tool to screen for anxiety disorder and its severity. Sum scores range from 0 to 21.

*Physical Complaints* Patients with depressive disorders often report an impaired physical state [42], in many cases bodily complaints are the most prominent symptoms. In this study, the subjective physical state was evaluated with a self-assessment questionnaire, the short version of Giessen Complaints Questionnaire (GBB-24) [43]. This well validated questionnaire consists of 24 items rated on a 5-point response scale. The dimensions “exhaustion”, “upper abdominal discomfort”, “aching joints and muscles”, “heart complaints” and a sum score for “pressure of complaints” were assessed. For a single dimension the scores range from 0 to 24, for the sum score from 0 to 96.

*Quality of life and mood* Quality of life was assessed with the Short Form Health Survey (SF-8) [44] which is a 8-item version of the SF-36 questionnaire measuring the subjectively perceived health-related quality of life and recording the overall subjective state of health of adults for different diseases, in relation to their physical, psychological, and social aspects. Summary measures for physical condition and mental well-being are calculated by weighting each SF-8 item using a norm-based scoring method. Higher summary scores indicate better health. Mood was assessed with the Berlin Mood Questionnaire, which contains 30 items assigned to dimensions of e.g. “elated mood”, “commitment”, “anxious depressiveness”, “tiredness”, and “apathy” [45]. Mean scores range from 0 to 4.

### Statistical analysis

Results are expressed as mean and standard deviation (SD) for parametric data and as median and quartiles for non-parametric data. Two-tailed student t-tests for normally distributed data and Mann–Whitney-U-tests for non-normally distributed data were used. Pearson’s coefficient of correlation was used to determine the degree of strength for the linear association between normally distributed variables and Spearman’s coefficient of correlation was used for non-parametric variables. Stepwise regression analyses were used to identify the strongest predictors of energy expenditure components and physical activity measures. Statistical significance was set at *P* < 0.05.

## Results

### Energy expenditure and patterns of physical activity

Total energy expenditure (TEE) was 3356 ± 562 kcal/d. Resting energy expenditure (REE) was 2180 ± 317 kcal/d according to the equation for obese individuals of Müller *et al.* [36]. Non-exercise AT (NEAT) was 707 ± 317 kcal/d and exercise-related AT (EAT) was in median 73 kcal/d (quartiles: 16/206). The components of AT adjusted to body weight were: non-exercise AT/kg 5.5 ± 2.8 kcal/kg/d and median exercise-related AT/kg 0.5 kcal/kg/d (quartiles: 0.1/1.4). The number of daily steps was 6206 ± 2681 and metabolic equivalent (MET) 1.07 ± 0.17. Duration of exercise-related AT was at median 5 min/d (quartiles: 2/15).

Participants with III° obesity compared to participants with II° obesity (see second and third part of Table 1) showed lower levels of body weight-adjusted NEAT, a lower average of MET, and a shorter daily duration of EAT. Contrary to our hypothesis, energy expenditure due to activity thermogenesis and patterns of physical activity did not differ between depressive and non-depressive patients. Patients with regular thyroid function (*n* = 32) or subclinical (mild) hypothyroidism (*n* = 3, thyroid-stimulating hormone [TSH]: 4.18, 5.96, and 8.74 mU/l) had a mean TSH of 2.30 ± 1.69 mU/l (reference range: 0.3–4.0 mU/l) compared to participants on replacement therapy with levothyroxine (*n* = 15) with a mean TSH of 1.73 ± 1.77 mU/l (two-tailed student *t*-test: *P* = 0.287). As expected, mean free thyroxine (fT4) was higher with 1.14 ± 0.19 ng/dl (reference range: 0.78–1.83 ng/dl) for participants on replacement therapy compared with a mean fT4 of 1.01 ± 0.20 ng/dl in patients with regular thyroid function or mild subclinical hypothyroidism (two-tailed student *t*-test: *P* = 0.026). Components of energy expenditure and patterns of physical activity did not differ between participants with regular thyroid function and patients on levothyroxine replacement therapy (see Table 1).

### Results in the psychosocial dimensions

By means of psychosomatic interviews 18 participants were clinically diagnosed to suffer from depression: in five cases a depressive episode (ICD-10: F32), one case of recurrent depressive disorder (ICD-10: F33), six cases of persistent affective disorder (dysthymia, ICD-10: F34.1), for one case “another affective disorder” (ICD-10: F38.8) was named, for one patient “mixed anxiety and depressive disorder” (ICD-10: F41.2) and for four patients “prolonged depressive reaction” due to an adjustment disorder (ICD-10: F43.2) were diagnosed. Eleven of these 18 participants were treated with (outpatient prescribed) antidepressants: one with opipramol, one with venlafaxine, three with citalopram, one with escitalopram, two with fluoxetine, two with sertraline, and one participant with paroxetine.

As depicted in Table 2, supporting the clinical diagnosis, patients with depression showed higher scores for depressiveness, psychosomatic co-morbidity (ISR-Scores for anxiety syndrome, obsessive syndrome, somatoform syndrome, supplementary scale, total score), higher anxiety score in GAD-7, more physical complaints (exhaustion, upper abdominal discomfort, aching joints and muscles, heart complaints, pressure of complaints), less mental well-being, less elated mood, and more negative mood like anxious depressiveness or tiredness. Coping strategies did not differ between patients with and without depression. Patients with obesity II° or III° did not differ in these psychosocial dimensions.

**Table 2:** Psychosocial dimensions

	Obesity		*P*	Depression		*P*
	II° (*n* = 9)	III° (*n* = 41)		No (*n* = 32)	Yes (*n* = 18)	
*Measurement of depressiveness (PHQ, ISR):*						
PHQ – Depression module	8.9 ± 3.6	10.1 ± 5.9	Ns	7.6 ± 3.8	14.0 ± 6.0	**
ISR - Depressive syndrome	1.3 (1.0/1.9)	1.3 (0.9/2.6)	Ns	1.3 (0.8/1.5)	2.4 (1.3/3.1)	**
*Measurement of coping strategies (Brief-COPE):*						
Avoidant coping	11.7 ± 3.3	13.4 ± 3.1 (*n* = 40)	Ns	12.8 ± 3.2	13.5 ± 3.2 (*n* = 17)	Ns
Support coping	11.9 ± 3.3	13.6 ± 3.2 (*n* = 40)	Ns	13.2 ± 3.3	13.4 ± 3.4 (*n* = 17)	Ns
Positive reframing	10.0 (8.5/17.0)	11.0 (10.0/12.8) (*n* = 40)	Ns	11.0 (10.0/13.8)	10.0 (9.0/11.5) (*n* = 17)	Ns
Active coping	11.1 ± 3.2	13.0 ± 2.8 (*n* = 40)	Ns	12.9 ± 3.0	12.1 ± 2.6 (*n* = 17)	Ns
*Measurement of additional psychosocial dimensions:*						
*Measurement of psychosomatic co-morbidity (ISR):*						
Anxiety syndrome	0.8 ± 0.9	1.1 ± 0.9	Ns	0.8 ± 0.7	1.6 ± 1.0	**
Eating disorder syndrome	2.6 ± 0.5	2.3 ± 0.8	Ns	2.2 ± 0.8	2.6 ± 0.6	Ns
Obsessive syndrome	0.0 (0.0/0.8)	0.0 (0.0/0.8)	Ns	0.0 (0.0/0.7)	0.7 (0.0/1.3)	*
Somatoform syndrome	0.7 (0.0/1.0)	0.0 (0.0/0.8)	Ns	0.0 (0.0/0.6)	0.7 (0.0/2.3)	*
Supplementary scale	0.7 ± 0.4	0.8 ± 0.5	Ns	0.6 ± 0.4	1.1 ± 0.5	***
Total score	1.0 ± 0.4	1.1 ± 0.6	Ns	0.8 ± 0.3	1.5 ± 0.6	***
*Measurement of anxiety (GAD-7):*						
Anxiety	7.7 ± 1.6	8.4 ± 5.4	Ns	6.8 ± 4.8	10.9 ± 4.2	**
*Measurement of physical complaints (GBB-24):*						
Exhaustion	11.1 ± 4.3	11.5 ± 5.8	Ns	9.7 ± 4.2	17.7 ± 6.1	**
Upper abdominal discomfort	5.2 ± 3.5	5.5 ± 4.0	Ns	3.8 ± 2.8	8.4 ± 4.0	***
Aching joints and muscles	13.0 ± 6.2	14.6 ± 4.7	Ns	12.8 ± 4.0	16.9 ± 5.6	*
Heart complaints	6.9 ± 3.9	6.5 ± 4.5	Ns	5.0 ± 3.3	9.4 ± 4.8	**
Pressure of complaints	36.2 ± 15.0	38.2 ± 16.1	Ns	31.3 ± 11.1	49.4 ± 16.5	***
*Measurement of quality of life and mood (SF-8, Berlin Mood Questionnaire):*						
SF-8: Total physical score	36.5 ± 9.4	32.3 ± 9.4 (*n* = 40)	Ns	33.9 ± 9.8 (*n* = 31)	31.6 ± 8.9	Ns
SF-8: Total mental score	43.1 ± 12.5	45.7 ± 13.2 (*n* = 40)	Ns	50.2 ± 10.4 (*n* = 31)	36.6 ± 12.8	**
Elated mood	1.4 ± 0.9	1.4 ± 0.9	Ns	1.7 ± 0.8	1.0 ± 0.8	*
Commitment	2.2 ± 0.9	2.3 ± 0.8	Ns	2.4 ± 0.8	2.1 ± 0.9	Ns
Anxious depressiveness	1.2 ± 0.9	1.5 ± 1.1	Ns	1.1 ± 0.9	2.1 ± 1.0	**
Tiredness	2.0 ± 0.5	1.9 ± 1.1	Ns	1.6 ± 0.8	2.4 ± 1.1	*
Apathy	0.2 (0.0/1.3)	0.4 (0.0/1.0)	Ns	0.3 (0.0/0.8)	0.4 (0.2/1.4)	Ns

*GAD-7* Generalized anxiety disorder scale, *GBB-24* Short version of Giessen Complaints Questionnaire, *ISR* ICD-10-Symptom-Rating questionnaire, *n* number of participants, *Ns* not significant, *PHQ* Patient Health Questionnaire, *SD* standard deviation, *SF-8*, Short Form Health Survey, *:*P* < 0.05, **:*P* < 0.01, ***:*P* < 0.001

Mean and standard deviation are given for parametric data, median and quartiles are given for non-parametric data. Two-tailed student t-tests were used for parametric data and Mann–Whitney-U-tests for non-parametric data

### Correlations of energy expenditure with age, body mass index (see Fig. 1) and psychosocial dimensions (see Table 3)

**Fig. 1:**
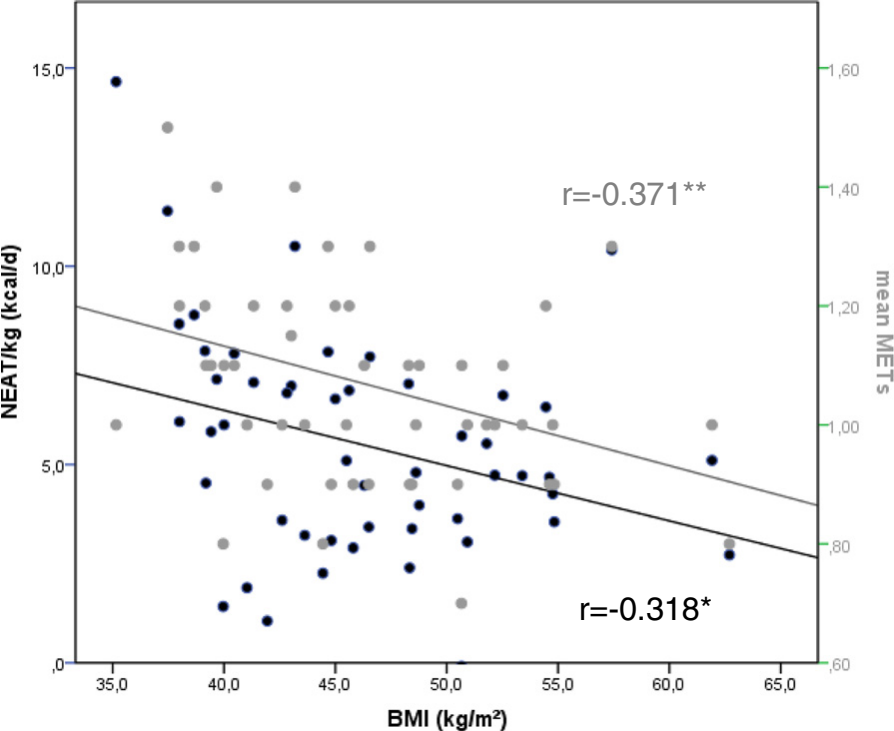
Pearson’s correlations coefficients for BMI with daily body weight-adjusted NEAT and mean MET. BMI: body mass index, MET: metabolic equivalent, NEAT: non-exercise activity thermogenesis, *:*P* < 0.05, **:*P* < 0.01

**Table 3:** Association (correlation coefficients) of psychosocial dimensions with age, BMI, components of energy expenditure and patterns of physical activity

		Activity thermogenesis		Physical activity		
		NEAT/kg	EAT/kg	steps/d	mean MET	Duration of EAT
*Demographic data:*						
Age (years)	r	−.114	−.093	−.436**	−.155	−.087
BMI (kg/m^2^)	r	−.318*	−.214	−.254	−.371**	−.225
*Depressiveness (PHQ, ISR):*						
PHQ - Depression module	r	.132	−.020	.023	−.009	−.055
ISR - Depressive syndrome	r	.114	−.138	−.018	−.026	−.158
*Coping strategies (Brief-COPE):*						
Avoidant coping	r	.008	−.088	.026	−.054	−.121
Support coping	r	−.336*	−.356*	.041	−.381**	−.356*
Positive reframing	r	−.191	.049	.081	−.180	.071
Active coping	r	−.363*	−.380**	−.078	−.395**	−.383**

*BMI* body mass index, *EAT* exercise-related activity thermogenesis, *EAT/kg* exercise-related activity thermogenesis/kg body weight, *ISR* ICD-10-Symptom-Rating questionnaire, *MET* metabolic equivalent, *NEAT/kg* non-exercise activity thermogenesis/kg body weight, *PHQ* Patient Health Questionnaire, *r* coefficient of correlation, *:*P* < 0.05, **:*P* < 0.01, ***:*P* < 0.001

Pearson’s coefficient of correlation was used for normally distributed variables and Spearman’s coefficient of correlation was used for non-parametric variables

Non-exercise AT and mean MET correlated negatively with body mass index (see Fig. 1), but did not correlate with depressiveness or the further psychosocial variables (psychosomatic co-morbidity, anxiety, physical complaints, quality of life and mood). The only psychological variables that correlated significantly with the physiological data here under investigation were the coping strategies “support coping” and “active coping”, correlating negatively with body weight-adjusted non-exercise AT (NEAT) and the parameters for physical activity: body weight-adjusted exercise-related AT (EAT), mean MET, and duration of exercise-related AT (see Table 3).

### Stepwise regression analyses of energy expenditure, age, body mass index and psychosocial dimensions

Due to intercorrelation of our variables we employed stepwise regression analysis and identified the coping strategy “active coping” as the most important predictor for body weight-adjusted NEAT (regression coefficient B −0.352, standard-error 0.132, *P* = 0.010), body weight-adjusted EAT (regression coefficient B −0.186, standard-error 0.066, *P* = 0.007), mean MET (regression coefficient B −0.020, standard-error 0.008, *P* = 0.015) and duration of EAT (regression coefficient B −1.748, standard-error 0.616, *P* = 0.007). In addition, mean MET was also predicted by BMI (regression coefficient B −0.008, standard-error 0.004, *P* = 0.026). For the number of daily steps the strongest predictor was age (regression coefficient B −104.228, standard-error 28.077, *P* = 0.001) followed by BMI (regression coefficient B −126.552, standard-error 52.605, *P* = 0.020).

## Discussion

In accordance with the above reviewed literature (given in the section “Introduction”) we assumed that a clinical diagnosis of depression or higher scores for depressiveness (assessed with validated questionnaires) and a dominance of passive (maladaptive) coping patterns would be associated with reduced AT and altered patterns of physical activity aggravating the degree of obesity. Furthermore, we assumed that these associations would be even more pronounced in a cohort of patients suffering from such an extent of obesity that they aim at a bariatric surgery procedure.

### Obesity and energy expenditure due to physical activity

In line with observations previously reported [7, 46] we found the expected differences between participants with II° or III° obesity for activity thermogenesis and patterns of physical activity: Patients with III° obesity spent significantly less energy in low- and moderate-intensity physical activities (weight-adjusted non-exercise AT), showed a significantly lower mean metabolic equivalent (MET), and spent less time in intensive physical activities (weight-adjusted exercise-related AT). Additionally we found that the higher BMI was, the lower were body weight-adjusted NEAT and mean METs.

### Depressive symptoms and energy expenditure due to physical activity

In contrast to reports of lower physical activity leading to or maintaining obesity in individuals suffering from depression [9, 16, 17], the hypothesised lower activity thermogenesis and altered physical activity patterns in patients with a diagnosis of (co-morbid) depression could not be proved.

An explanation for these conflicting results might be the difficulty of assessing energy expenditure: The abovementioned studies used physical activity recall questionnaires or interviews for quantifying physical activity in daily life [9, 17]. The inaccuracy of these activity logs is a major limitation in this approach. Four different questionnaires showed deviations up to 60 % in comparison with the doubly labelled water method for the evaluation of physical activity [32]. In particular, obese participants more than normal weight individuals tend to overestimate their activity. Prince *et al.* addresses poor correlations between self-report and direct measures of physical activity [33]. Motion sensors including simple pedometers (measurement of steps) and technologically more advanced accelerometers (detection of body acceleration) provide objective approaches to assess physical activity. Tucker and Earl assessed emotional health (with the General Well-Being Schedule), energy intake (with food records) and physical activity using accelerometers (Actigraph™ accelerometer) during a follow-up period of approximately 2 years in middle-aged women [47] and found that women with lower scores of emotional health seem to have a greater risk of weight gain. However, after adjustment for potential confounders this risk is not greater in depressed women than in their non-depressed counterparts.

We assume a high validity of our data concerning AT and physical activity patterns assessed with the portable armband device. However, one limitation might be that the time period of measurement might be too short as a distinct variation of physical activity has been described across the whole week with a decrease of moderate physical activity on Saturdays with rising weight category [34]. In order to overcome this limitation, we assessed energy expenditure preferably on 2 weekdays and 1 weekend day.

Another limiting aspect for interpretation of our data is the high percentage of depressed participants treated with antidepressants (11 out of 18). Although participants with clinically diagnosed depression still show higher scores for depressiveness, we cannot exclude that the treatment with antidepressant drugs attenuates the hypothesised correlation between physical activity and depression.

Since we studied only individuals with high-grade obesity one might speculate that the decreases in AT and physical activity associated with body mass are so strong that they outweigh possible changes in energy expenditure associated with depression, which have been described before only in overweight individuals or those with low-grade obesity. This explanation is corroborated by findings of Hrabosky *et al.* in obese persons suffering from binge eating disorder [48]. Physical inactivity assessed with a self-report questionnaire (Paffenbarger Physical Activity Questionnaire) is correlated with BMI (mean BMI 38.2 ± 5.7 kg/m^2^) but not with the depressive affect as measured with the Beck Depression Inventory [48].

### Coping strategies and activity thermogenesis

Rydén *et al.* described lower problem-focused and higher emotion-focused coping scores in candidates for bariatric surgery compared to individuals preferring a conventional treatment [31]. Similar results were reported by Hörchner *et al.* [49]. In our cohort higher scores for the supposed adaptive coping strategies “support coping” and “active coping” were accompanied by lower body weight-adjusted NEAT, lower exercise-related AT, lower mean MET, and shorter duration of exercise-related AT.

In the original version of the Brief-COPE the scale “active coping” was constructed by Carver as a problem-oriented approach to difficult situations [28]. However, for our cohort the scale “active coping” does not seem to assess problem-focused coping in the sense of leading to a necessary increase of physical activity. The “active coping” scale includes the following items: “I’ve been concentrating my efforts on doing something about the situation I’m in.“;”I’ve been taking action to try to make the situation better”; “I’ve been thinking hard about what steps to take.”; and “I’ve been trying to come up with a strategy about what to do.”. The high scores may be explained by our patients’ perception of being very active to achieve approval for bariatric surgery (e.g. participation in patient information events and support group meetings, preparation and submission of applications for cost coverage of bariatric surgery to the patients’ health insurance company). This is in line with the finding recently described by Ahnis *et al.* [50] that patients prior to bariatric surgery showed higher scores for “active coping” (Brief-COPE) than obese patients participating in a 1-year multimodal outpatient weight reduction program.

## Conclusions

We found that lower energy expenditure due to physical activity is associated with higher grades of obesity. However, our assumption of lower activity thermogenesis and of differences in physical activity patterns (like a lower number of daily steps, and a lower value of mean MET) in depressive obese individuals could not be confirmed. The coping strategies “active coping” and “support coping” - interpreted in the sense of partly delegating the task of weight management to surgeons - were associated with lower body weight-adjusted activity thermogenesis.

Our findings highlight the enormous obstacles for high-grade obese patients to increase their physical activity which would be necessary for conservative achievement of long-lasting weight loss indicating bariatric surgery to be a more promising treatment option. These obstacles were less clearly associated with affective disturbances than was expected from reports in the literature (concerning degree of depressiveness, psychosomatic co-morbidity, anxiety, physical complaints, and quality of life and mood). However, it might be that the patients’ decision to aim at bariatric surgery leads to under-reporting of affective and behavioural issues which might jeopardize their success in obtaining the surgical procedure.

## Abbreviations

AT: Activity thermogenesis
BMI: Body mass index
EAT: Exercise-related activity thermogenesis
MET: Metabolic equivalent
NEAT: Non-exercise activity thermogenesis
REE: Resting energy expenditure
TEE: Total energy expenditure
